# A causal role for TRESK loss of function in migraine mechanisms

**DOI:** 10.1093/brain/awz342

**Published:** 2019-11-19

**Authors:** Philippa Pettingill, Greg A Weir, Tina Wei, Yukyee Wu, Grace Flower, Tatjana Lalic, Adam Handel, Galbha Duggal, Satyan Chintawar, Jonathan Cheung, Kanisa Arunasalam, Elizabeth Couper, Larisa M Haupt, Lyn R Griffiths, Andrew Bassett, Sally A Cowley, M Zameel Cader

**Affiliations:** 1 Translational Molecular Neuroscience Group, Weatherall Institute of Molecular Medicine, Nuffield Department of Clinical Neurosciences, University of Oxford, Oxford, UK; 2 Institute of Neuroscience and Psychology, College of Medical, Veterinary and Life Sciences, University of Glasgow, Glasgow, G12 8QQ, UK; 3 Department of Clinical Neurology, Nuffield Department of Clinical Neurosciences, University of Oxford, Oxford, UK; 4 James Martin Stem Cell Facility, Sir William Dunn School of Pathology, University of Oxford, Oxford, UK; 5 Genomics Research Centre, Institute of Health and Biomedical Innovation, School of Biomedical Sciences, Queensland University of Technology, Brisbane, QLD 4059, Australia; 6 Wellcome Trust Sanger Institute, Wellcome Genome Campus, Hinxton, Cambridge, CB10 1SA, UK

**Keywords:** TRESK, migraine, induced pluripotent stem cells, nociceptors, GTN

## Abstract

The two-pore potassium channel, TRESK has been implicated in nociception and pain disorders. We have for the first time investigated TRESK function in human nociceptive neurons using induced pluripotent stem cell-based models. Nociceptors from migraine patients with the F139WfsX2 mutation show loss of functional TRESK at the membrane, with a corresponding significant increase in neuronal excitability. Furthermore, using CRISPR-Cas9 engineering to correct the F139WfsX2 mutation, we show a reversal of the heightened neuronal excitability, linking the phenotype to the mutation. In contrast we find no change in excitability in induced pluripotent stem cell derived nociceptors with the C110R mutation and preserved TRESK current; thereby confirming that only the frameshift mutation is associated with loss of function and a migraine relevant cellular phenotype. We then demonstrate the importance of TRESK to pain states by showing that the TRESK activator, cloxyquin, can reduce the spontaneous firing of nociceptors in an *in vitro* human pain model. Using the chronic nitroglycerine rodent migraine model, we demonstrate that mice lacking TRESK develop exaggerated nitroglycerine-induced mechanical and thermal hyperalgesia, and furthermore, show that cloxyquin conversely is able to prevent sensitization. Collectively, our findings provide evidence for a role of TRESK in migraine pathogenesis and its suitability as a therapeutic target.

## Teaser

The two-pore potassium channel TRESK is a potential drug target in pain and migraine. Pettingill *et al.* show that the F139WfsX2 mutation causes TRESK loss of function and hyperexcitability in nociceptors derived from iPSCs of patients with migraine. Cloxyquin, a TRESK activator, reverses migraine-relevant phenotypes *in vitro* and *in vivo*.

## Introduction

The two-pore domain K^+^ channel (K2P) family mediate ‘leak’ potassium current, which is a major contributor to neuronal resting membrane potential and also serves to counter depolarizing stimuli. TRESK is a K2P channel with enriched expression in sensory ganglia with several lines of evidence implicating channel activity in normal and pathological pain processing. TRESK knockout mouse models demonstrate increased excitability of sensory ganglia and behavioural hypersensitivity to noxious and non-noxious sensory stimuli, consistent with an inhibitory effect of TRESK on neural excitability (Dobler *et al.*, 2007; Weir *et al.*, 2019). Pharmacological inhibition of TRESK induces nocifensive behaviours in healthy rats (Tulleuda *et al.*, 2011). In the context of pathological pain, *KCNK18* (TRESK) mRNA is downregulated following nerve injury and delivery of exogenous TRESK ameliorates pain hypersensitivity following nerve injury, consistent with a crucial role in neuropathic pain development/maintenance (Tulleuda *et al.*, 2011; Zhou *et al.*, 2013). This has prompted several drug discovery efforts, with TRESK activators seen as a promising strategy for analgesic drug development (Wright *et al.*, 2013; Bruner *et al.*, 2014). However, a confound of pursuing drug discovery based on these studies is that human and mouse TRESK demonstrate significant differences, including low sequence homology and differing pH sensitivity (Keshavaprasad *et al.*, 2005).

The role of *KCNK18*, the gene encoding the TRESK K2P channel, in genetic causation of migraine has been more controversial. A frameshift mutation, F139WfsX24 in *KCNK18* was originally identified in one migraine proband, as part of a larger study investigating the role of brain-expressed ion channel genes in paroxysmal neurological disorders, wherein 150 ion channel genes were sequenced in 110 unrelated migraine subjects (Lafreniere *et al.*, 2010; Lafreniere and Rouleau, 2012). The migraine proband belonged to a large multi-generation family segregating migraine with aura in an autosomal dominant pattern, and a genome-wide linkage screen in this family showed significant linkage on chromosome 10q25.2-3, a region harbouring *KCNK18 *(Lafreniere *et al.*, 2010). The frameshift mutation, which prematurely truncates TRESK, furthermore caused a dominant negative loss of TRESK function of the channel when expressed in *Xenopus* oocytes. In trigeminal ganglion, TRESK is the dominant K2P channel (Bautista *et al.*, 2008) and the collective data were supportive that TRESK may have an important role in migraine pathogenesis.

However, in further work, sequencing of *KCNK18* in 511 sporadic migraine subjects and 505 ethnically-matched controls revealed five further missense variants and no frameshift mutations in this cohort (Andres-Enguix *et al.*, 2012). Of these missense mutations, only A34V (one migraine proband alone) and C110R (present in migraineurs and controls) caused a loss of function in *Xenopus* oocytes. Furthermore, no other migraine families have been reported with segregating deleterious mutations in *KCNK18*; there is no evidence of genetic association in sporadic migraine and the ExAC browser identifies a number of potentially disruptive mutations including frameshift mutations in *KCNK18 *(Lek *et al.*, 2016) that are present in the general population. Overall, despite the genome-wide significant linkage in the originally reported family, the evidence for a role of gene mutations in *KCNK18* in migraine genetic causation is weak.

We therefore sought to investigate the effects of the F139WfsX24 mutation on human nociceptor function by generating induced pluripotent stem cell (iPSC) lines from three migraine with aura affected female siblings from the originally described family. This allowed us to study the functional effects of the mutation in its native cellular and genetic environment. CRISPR-Cas9 genome engineering also afforded us the opportunity to correlate cellular phenotypes to the F139WfsX24 allele. We find that TRESK is expressed in human non-peptidergic nociceptive neurons differentiated from iPSCs and that the F139WfsX24 mutation leads to complete loss of TRESK current with resulting heightened neuronal excitability. These results are in contrast to the normal excitability state of C110R iPSC nociceptors, offering insight as to why the two variants may have differing disease penetrance. We then show the potential role of TRESK activators as a pain and migraine therapeutic by demonstrating that cloxyquin can ameliorate phenotypes of an *in vitro* pain model and the chronic nitroglycerine (GTN) *in vivo* migraine model. Our work therefore demonstrates the importance of TRESK in human nociceptor function and in migraine mechanisms.

## Materials and methods

### Ethics statement

#### Induced pluripotent stem cells

The human iPSC lines used for this study were derived from human skin biopsy fibroblasts and blood erythroblasts, following signed informed consent, with approval from the UK NHS Research Ethics Committee (REC: 13/SC/0179 and 10/H0505/71) and were derived as part of the IMI-EU sponsored StemBANCC consortium. Fibroblasts for line SBAD2 and NHDF1 were purchased from Lonza, who provide the following ethics statement: ‘These cells were isolated from donated human tissue after obtaining permission for their use in research applications by informed consent or legal authorization’. The C110R lines were derived from erythroblasts obtained from a subject recruited in QUT, Australia (HREC: 1400000748).

#### Animal studies

All procedures were carried out under an approved UK Home Office License, in accordance with the UK Home Office Animals (Scientific Procedures) Act 1986. Every effort was made to minimize animal numbers and suffering by adhering to the ARRIVE guidelines. Mice were housed in pathogen-free individually ventilated cages with 12:12-h light/dark cycles and access to food and water was given *ad libitum*. Animals were housed in cages of not more than five animals, between 2 and 4 months old and weighing 20–30 g over the course of the testing.

### Exome analysis

DNA was prepared for exome sequencing using the Nimblegen SeqCap EZ Exome v3.0 kit according to the manufacturer’s instructions. Libraries were sequenced on the Illumina HiSeq 4000 platform, producing 75-bp paired-end reads. The equivalent of ∼60M 75-bp read-pairs were generated for each sample. Reads were mapped to the reference genome (hs37d5) using Stampy and PCR duplicates were removed using Picard. Variants were called within the bait regions ± 50 bp using Platypus and annotated with a number of databases by a combination of Variant Effect Predictor (VEP) and in-house Python scripts. AVIA v2.0 was used to annotate sequencing variants (Vuong *et al.*, 2015). Calculation of maximum allele frequency of a disease causing variant in migraine was performed using alleleFrequencyApp (Whiffin *et al.*, 2017), with population prevalence of 1:7, a maximum proportion of Mendelian genetic cases of 10% and a penetrance of 1.

### Generation and culture of iPSC lines

In this study, we used nine iPSC-derived lines outlined in Supplementary Table 1. Newly derived lines were reprogrammed using the Nakanishi SeVdp vector Sendai system described previously (Nishimura *et al.*, 2011) or CytoTune® (Life Technologies). Six lines used were derived from skin biopsies: three healthy controls (AH017, SBAD02, NHDF1) and three lines from migraine patients (837, 838, 839), three sisters all belonging to the same family with the F139WfsX24 mutation in *KCNK18* gene encoding TRESK. Two additional lines were reprogrammed from blood erythroblasts; BPC345 (healthy control) and BP8512 (containing the C110R TRESK variant). A further iPSC line, RCi002-A, which is from a patient with inherited erythromelalgia and having the F1449V mutation in SCN9A, was obtained from the EBiSC consortium and has been published (Cao *et al.*, 2016).

### Assessment of genome integrity

Before initiation of differentiation, iPSCs genome integrity was assessed by an Illumina HumanCytoSNP-12v2.1 beadchip array (∼300 000 markers) and analysed using KaryoStudio software (Illumina). Single nucleotide polymorphism (SNP) deviations in iPSC lines were compared to parent fibroblast pools. IPSC pluripotency was assessed for pluripotency markers NANOG and OCT-4 by immunocytochemistry.

To confirm genotype identity of iPSC lines, genomic DNA was isolated from patient and control iPSCs and polymerase chain reaction (PCR) was performed with primers flanking exon 3 (Forward 5′-TGTCATGCCAAGGAG-3′, Reverse 5′-GATAAGATGGTTGCCAG-3′) of the *KCNK18* gene containing the F139FsX39 mutation and the amplicon Sanger sequenced.

IPSCs were cultured in feeder-free conditions on growth-factor-reduced Matrigel® (Corning)-coated tissue culture dishes, and fed daily with mTeSR1 (StemCell Technologies). Cells were passaged as patches every 4–7 days, upon reaching 80–95% confluency, using 0.5 mM EDTA in phosphate-buffered saline (PBS, Life Technologies).

### Generation of isogenic human TRESK iPSC lines with CRISPR/Cas9 gene-editing technology

For targeting *KCNK18* by CRISPR/Cas9 we designed two guide RNAs (gRNAs) in http://crispr.mit.edu to target the F139WfsX24 mutant allele of *KCNK18* (*KCNK18* px462#TRESK1 5′-ACTTGCCAAGCCTGGTGACGGGG-3′ and *KCNK18* px642TRESK#2 5′-TGTGCATGCTCTATGCTCTTTGG-3′) using a paired nickase strategy (Ran *et al.*, 2013). Guides were cloned into the dual Cas9 and gRNA-expressing, puromycin-resistant pX462 plasmid (Addgene #48141, a kind gift of F. Zhang).

For gene editing at the F139WfsX24 locus, iPSCs from migraine line 839 were passaged in to single cells and 10^6^ iPSCs were electroporated (1000 V, 40-ms pulse width, one pulse) with 5 μg of both donor plasmids using Neon (Invitrogen) and 1 μl of 10 μM single stranded oligonucleotide donor (ssODN) template of wild-type TRESK containing 100 bp arms flanking either side of the F139WfsX24 mutation (Ultramer, IDT technologies). After electroporation, iPSCs were plated at high density (5 × 10^5^ cells/cm^2^). At 1 day post-transfection, puromycin (1 μg/ml, Life Technologies) selection was performed for 48 h, and surviving iPSCs were subsequently plated at low density onto mitotically-inactivated mouse embryonic feeder [MEF from CF1 mice (Millipore)]; cells on gelatin-coated tissue culture plates in human embryonic stem cell medium [KnockOut DMEM, 2 mmol/l l-glutamine, 100 mmol/l non-essential amino acids, 20% serum replacement, and 8 ng/ml basic fibroblastic growth factor (FGF2)]. Individual colonies were manually selected for expansion and converted to growth on Matrigel® in mTeSR™1. Genomic DNA was isolated, and colonies genotyped using *KCNK18*-specific PCR primers (above) and Sanger sequencing to confirm correction of F319WfsX24 mutation to the wild-type sequence. The CRISPR correction introduced a silent mutation p(Prol142) 3 bp downstream. Gene edited lines were assessed and analysed using KaryoStudio and GenomeStudio software. SNP deviations in iPSC lines were compared to parent fibroblast pools.

### Nociceptor differentiation

IPSC-derived nociceptors were generated using previously published protocols (Chambers *et al.*, 2012). Briefly, iPSCs were treated with accutase to produce single cells plated at a density of 40 000 cells/cm^2^ and plated with the Rho-kinase inhibitor Y-27632 (10 μmol/l; Abcam). Neuralization was initiated when iPSCs were confluent using MEF-conditioned KSR media (Knockout DMEM, 15% knockout serum replacement, 1% non-essential amino acids, 1% GlutaMAX^TM^, 1% penicillin/streptomycin and 100 μM β-mercaptoethanol) media supplemented with 100 nM LDN-193189 and 10 μM SB431542 to inhibit SMAD signalling. Cells were fed daily and N2 media (Neurobasal® medium, N2 supplement, B-27^TM^ supplement without vitamin A, GlutaMAX^TM^, penicillin/streptomycin) added in 25% increments every other day starting on Day 4. Supplementation of three inhibitors, 3 μM CHIR99021, 10 μM SU5402 and 10 μM DAPT, on Days 2–10 initiated nociceptor induction. On Day 12 cells were treated with accutase, replated as single cells and maintained in long-term culture in N2 media supplemented with human-b-NGF (25 ng/ml), BDNF (20 ng/ml) and GDNF (20 ng/ml) to promote maturation. The differentiated nociceptors were cultured for an additional 50 days before functional assays were performed. For each experiment, iPSCs were independently differentiated into nociceptors at least three times

### Electrophysiology

Coverslips containing iPSC‐derived nociceptors were placed in a recording chamber mounted onto the stage of an upright microscope and the soma were targeted for. Cells were continuously superfused with oxygenated artificial CSF (95% O_2_/5% CO_2_) containing 140 mM NaCl, 4.7 mM KCl, 2.5 mM CaCl_2_, 1.2 mM MgCl_2_, 10 mM HEPES and 10 mM glucose (pH 7.3). Patch‐clamp electrodes (4–7 MΩ) were filled with an intracellular solution containing 130 mM KCl, 1 mM MgCl_2_, 5 mM MgATP, 10 mM HEPES and 0.5 mM EGTA; pH 7.3. Recordings were obtained using a Multiclamp 700B amplifier and digitized at 10–20 kHz using Digidata 1550 acquisition board and data analysed with Clampfit 10 (Molecular Devices). Cell capacitance was compensated for by the circuitry of the amplifier and series resistance was compensated 60–80% to reduce voltage errors. Upon establishing whole-cell access, baseline biophysical properties such as cell capacitance, resting membrane resistance and input resistance were measured and monitored throughout. Cells were abandoned if values exceeded −50 mV. Sodium and potassium currents were evoked by voltage steps ranging from −80 to +50 mV in 10-mV increments. For current-clamp recordings, voltage responses were evoked from a holding potential of −60 mV using 50-ms steps ranging from 0 to +400 pA in 10-pA intervals, until an action potential was elicited. Repetitive firing was defined as those cells firing more than one action potential to a prolonged (500 ms) supra-threshold depolarizing current injection (twice the rheobase current). The experimenter was not blinded to genotype for patch clamp experiments.

### TPA inhibition of TRESK

We used a previously reported ramp protocol to dissect TRESK currents (Dobler *et al.*, 2007; Liu *et al.*, 2013). In voltage-clamp, nociceptors were held at −60 mV before depolarization to −25 mV for 300 ms and then subsequently ramped to −135 mV over 550 ms. Outward currents were measured at the end of the 300-ms step. Baseline measurements were collected in the presence of 1 μM tetrodotoxin (TTX) and subsequently nociceptors perfused with 500 nM tetrapentylammonium bromide (TPA, Sigma 241970) in the presence of TTX for 20 min before reassessing TRESK currents.

### Multi-electrode array culture

IPSC lines were differentiated to nociceptors and plated on Matrigel®-coated MEA (multi-electrode array) plates (Cytoview MEA 48, Axion Biosystems) at Day 12 post-induction with dotting technique at density of 156 000/cm^2^. At Day 14 the DRG culture was treated for 24 h with 4 µM Cytosine-β-arabinofuranoside (C1768, Sigma). At Day 16, 52 000/cm^2^ rat astrocytes (N7745-100, Thermo Fisher) were added to the culture to support neuronal health and attachment. The rat astrocyte co-culture does not interfere with the neuronal properties that we measured (Supplementary Fig. 2), although an extensive characterization with and without astrocytes was not performed. Half of the growth media in the culture was changed every 2 to 3 days, supplemented with laminin (L2020, Sigma, 1:1000 dilution) once a week. MEA cultures were kept at 37°C and 5% CO_2_ level. The effects of cloxyquin and TPA were investigated on Days 60–65 of SCN9A (Na_v_1.7) IPSC-derived nociceptors with replicates of six wells for a single condition.

For MEA recordings, 2-min recordings were taken after 5-min plate settling time on the MEA reader. The plate was kept at 37°C while recordings were taken. For drug incubation experiment, activity was recorded for 2 min as baseline before cloxyquin and TPA were added sequentially. Drug experiments were performed at time points where the spontaneous activity was stable for at least 3 h before experiment.

### Multi-electrode array data analysis

Mean firing rates were calculated using AxIs software (Axion Biosystems). MEA records the extracellular spiking activity of neurons. Spikes were detected online by AxIS spike detector software as described previously (McConnell *et al.*, 2012) and were defined as an event with amplitude >6 standard deviations (SD) above background noise. Single-electrode bursts were defined as at least five consecutive spikes with an interspike interval <100 ms. Following recording, spike sorting was performed using the AxISmetrics plotting tool (Axion Biosystems). Raster plots with histograms were plotted with NeuralMetric Tools. Raw data scatter plot and percentage change analysis was performed with custom built program in MATLAB using mean firing rate information extracted from raw data from AxIS. In the analysis of drug effect graph, after excluding inactive electrodes (<5 spikes/s), all data from electrodes from the six-well replicates of a condition was pooled together. Electrodes with firing frequency that fell in-between 25th and 75th percentile in the baseline reading before drug application were used for analysing effects post drug-addition.

### Immunocytochemistry

IPSC-derived nociceptors were fixed with 4% paraformaldehyde-PBS for 20 min, washed, permeabilized with 0.1% Triton™ X-100 and incubated with blocking solution for 1 h at room temperature. Cells were subsequently incubated overnight at 4°C with antibodies to pluripotency (anti-Oct4, 1:200, ab19857), neural crest (p75^NTR^, 1:100, Promega, G323A) and neuronal markers (anti­TUJ1, 1:500, MMS-435P, Covance; anti-Islet-1, 1:100, DSHB-394D5.s; anti-Ret, 1:100, Alamone; and anti-TRESK 1:100 sc-51238 Santa Cruz). Cells were washed, and incubated with Alexa Fluor® 488/568 conjugated antibodies (Life Technologies) for 1 h. Nuclei were stained with DAPI and fluorescence visualized using confocal microscopy with a Zeiss 880 inverted microscope.

Efficiency of neuronal differentiation was assessed by counting the number of TUJ1+ neuronal cells expressing sensory neuronal marker Islet-1 and Brn3a. Immunocytochemistry TRESK expression was analysed measuring the pixel intensity counted using ImageJ software.

### Immunoblotting

For cell surface expression studies, iPSC-derived nociceptors were grown in 6 cm^2^ dishes. On Day 50 cells were washed, solubilized in fractionation buffer (20 mM HEPES, 10 mM KCl, 2 mM MgCl_2_, 1 mM EDTA, 1 mM EGTA, 1 mM DTT and protease inhibitors), and passed through a 27G needle. Samples were centrifuged (3000 rpm and subsequently 8000 rpm) and cytoplasm and mitochondria fractions discarded. The membrane fraction was collected by centrifuging the sample at 40 000 rpm. Equal amounts of SDS sample buffer were added and 10 μg samples were resolved on a Tris-glycine (3–8%) gel (NuPAGE, Invitrogen). Western blots were blocked (5% milk in 1% PBS-Tween) and probed with a polyclonal TRESK antibody (sc-51238 P-19, SantaCruz) overnight. Antibody to the transferrin receptor (1:500, 13-6800, Invitrogen) was used as a cell surface fraction loading control followed by anti- rabbit and anti-mouse HRP-conjugated secondary antibodies (1:2000, P0448 and P0447 DAKO) and visualized using ECL (Pierce #32106).

### Quantitative RT-PCR

Total RNA from cultured cells was extracted using an RNeasy® Mini kit (Qiagen) and 300 ng of RNA was reverse-transcribed using SuperScript™ II (Invitrogen). Quantitative PCR was performed by reverse transcription with SYBR® Green PCR Master Mix (Invitrogen) using an Applied Biosystems® 7500 Real-Time qPCR machine. Gene expression is reported as relative quantities using the ΔΔCT method. All gene expression values were normalized to the housekeeping gene, *GAPDH*. Gene expression primers were as follows: *MET*/c-Met (F primer 5′-ACCTTTGATATAACTGTTTACTTGTTGCA-3′, R primer 5′-GCTTTAGGGTGCCAGCATTTT-3′); *RUNX1* (F primer 5′-CAAGACCCTGCCCATCGCTT-3′, R primer 5′-GTAGTTTTCATCATTGCCAGCC-3′); *KCNK18*/TRESK (F primer 5′-GTGTTCAGCACCGTGGGCTA-3′, R primer 5′-CCGTGAGAACGAGGAACATCA-3′); and *GAPDH* (F primer 5′-AGCCACATCGCTCAGACAC-3′, R primer 5′-GCCCAATACGACCAAATCT-3′).

### Animals

Two- to four-month-old mice on a C57BL/6J background (Charles River, UK) were allocated to treatment groups by randomly drawing them from their cages and rehousing post-treatment in mixed treatment cages. Sample sizes for the different behavioural outcomes were based on previous literature, seeking to observe a 25% change, whereby experiments were performed with a group size of 5–12 animals (Pradhan *et al.*, 2014; Farkas *et al.*, 2016; Tipton *et al.*, 2016). TRESK knockout mice were compared to littermate wild-type mice in all studies. TRESK knockout animals were generated by the trans-NIH Knock-Out Mouse Project (KOMP, www.komp.org). In brief, the VelociGene targeting system was used to ablate *Kcnk18* by replacing the majority of the coding region with a LacZ-neomycin cassette.

### Nitroglycerine mouse model

GTN (Hameln) was prepared for injection as a 1 mg/ml solution reconstituted in saline and injected following baseline behavioural testing and 2 h prior to repeat testing. Cloxyquin (Sigma) was reconstituted in 3.6% DMSO in DPBS and given at 10 mg/kg and 50 mg/kg as intraperitoneal injections at the same time as GTN. Control animals received the appropriate vehicle (saline or 3.6% DMSO in DPBS) in all comparative behavioural tests.

#### Von-Frey

Static mechanical thresholds in freely moving awake mice were examined via von Frey hair application (0.008–1g; Touch Test™ Stoelting) to the plantar surface of the hindpaw using the ‘up–down’ method (Chaplan *et al.*, 1994). Before testing, mice were randomly assigned in to individual plexiglass cubicles (8 × 5 × 10 cm) for 1 h on an elevated wire mesh floor to enable access to the paw surface. Calibrated von Frey hairs were applied to the left and right hind paws, starting with the 0.6g filament until the fibre bowed. A positive withdrawal response is followed by a lower force hair and vice versa for a negative withdrawal response until a change in behaviour occurs. Using this up–down sequence, four subsequent hairs were assessed and the 50% paw-withdrawal threshold was calculated as described by Dixon (1980). Data presented are from both left and right hind paws averaged together as no laterality effects were observed.

#### Hargreaves

Thermal thresholds in freely-moving awake mice were assessed with the Hargreaves method using the plantar test (37370, Ugo Basile). Before testing, mice were assigned to testing cubicles at random and habituated to the apparatus for 1 h in individual cubicles (8 × 5 × 10 cm) placed on a glass plate. An infrared light source was applied to the plantar surface of hindpaws through the glass plate. Withdrawal reflexes were recorded from both left and right paws, on three occasions, leaving at least 2 min between stimuli.

#### Hot plate

Noxious-thermal thresholds of the hindpaws were examined via the hot plate test using an incremental hot/cold plate (35150, Ugo Basile) set at a temperature of 50 and 53°C ± 0.2°C. Mice were placed in the centre of the hot plate in a 10-cm diameter testing box; licking, jumping or stamping reflex responses were recorded to the nearest 0.01 s. A maximum latency of 20 s was permitted to prevent tissue damage.

#### Orofacial pain stimulation testing

Mice were placed in a 15–30 g head access restraining cylinder (Stoelting Co.) and acclimatized for 5–10 min. The restrainer allowed access to the head through various holes, with minimal ability for the mouse to move. Testing began when the mouse stopped exploring the cylinder. A von Frey filament of 0.4g force was applied to the periorbital region, until the filament bent (Farkas *et al.*, 2016). The filament was applied three times and responses were recorded using a descriptive scoring system as follows: no response (0 point), mild response: gentle head shake/ forepaw swipe (0.25 points), moderate response: vigorous head shake/aggression to filament (0.50) and obvious response: complete withdrawal of the head (1 point). The points were combined to give a complete orofacial pain response score whereby a score of 3 is the highest response to record.

### Statistical analysis for animal studies

The effect of an acute single GTN dose was assessed using an unpaired *t*-test, or repeated measures one-way ANOVA followed by a Tukey’s multiple comparisons test in Prism (GraphPad). For analysis of baseline trends in sensory responses and comparison of sensory responses between wild-type and TRESK knockout after repeated GTN injections, a two-way ANOVA with Tukey’s multiple comparisons test was used. *P*-values of <0.05 were considered significant in all tests. The operator was blind to genotype and treatment when performing the assays.

### Data availability

Raw data were generated at the NDCN, University of Oxford. Derived data supporting the findings of this study are available from the corresponding author on request.

## Results

### Exome analysis of *KCNK18* F139WfsX24 patients

The original finding of a *KCNK18* frameshift mutation in a migraine pedigree supported by genome-wide significant linkage for the region containing *KCNK18*, held promise that a rare variant cause of typical migraine had been identified. The original linkage region was located on chromosome 10q25.2-3 and as the linkage region was broad, several other genes could also explain the linkage signal. To investigate this further, in three affected individuals from the *KCNK18* F139WfsX24 family, whole exome sequencing was performed with an average 100-fold coverage per exome. This identified 41 exonic SNPs, three exonic indels and four exonic substitutions shared by all three individuals in the linkage region. The F139WfsX24 mutation in *KCNK18* was found in all three affected individuals. Using an established statistical-based method for frequency-based filtering of candidate monogenic disease-causing variants, which accounts for disease prevalence, genetic and allelic heterogeneity, inheritance mode and penetrance, we calculated an expected maximum allele frequency in the general population for a potential disease-causing migraine variant (0.7%) (Whiffin *et al.*, 2017). Of the variants identified in the linkage region by exome sequencing only the F139WfsX24 *KCNK18* mutation was found at a lower frequency than this threshold in the ExAC cohort. This indicates that the F139WfsX24 *KCNK18* mutation is most likely responsible for the genetic linkage in this family.

### TRESK F139WfsX24 and control pluripotent stem lines differentiate efficiently into mature non-peptidergic nociceptors

After establishing that the *KCNK18* frameshift mutation was likely to be responsible for the genetic linkage in this family, we sought to investigate whether the mutation would lead to changes in human cellular physiology relevant to migraine headache susceptibility. While the cellular mechanisms of migraine remain poorly understood, headache arises from activation of nociceptive neurons of the trigeminal ganglion that innervate dural blood vessels. We therefore used a previously reported method to produce functionally mature human peripheral nociceptors (Chambers *et al.*, 2012). We generated iPSC lines from dermal fibroblasts using non-integrating Sendai virus from three related migraine with aura patients with the TRESK F139WfsX24 mutation and compared them to three unrelated healthy control subjects (Hartfield *et al.*, 2014; Handel *et al.*, 2016) who had not reported a significant headache disorder (Supplementary Table 1). We confirmed successful reprogramming by immunostaining for pluripotency markers NANOG and OCT-4, and genomic integrity by SNP karyotyping (Supplementary Fig. 1).

**Figure 1:**
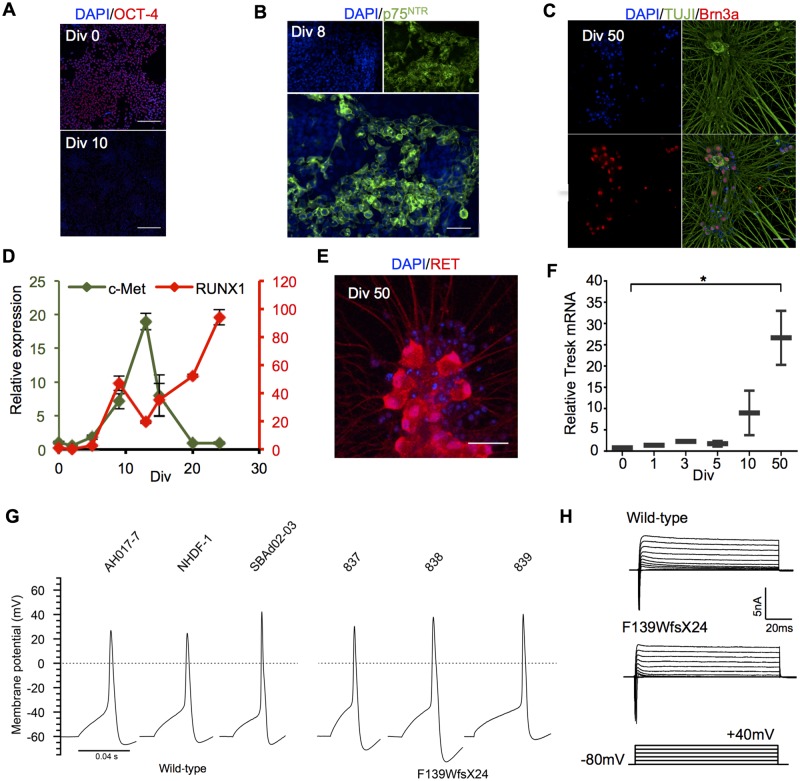
**Differentiation of nociceptors from iPSCs.** (**A**) Representative image of iPSC line losing the pluripotency marker, OCT-4 expression during nociceptor differentiation. Scale bar = 200 µm. (**B**) Widespread protein expression of the neural crest marker p75(NTR) at Day 8. (**C**) Representative images illustrating the presence of neuronal (TUJ1) and sensory (Brn3a) markers in mature neurons after 50 days in culture. (**D**) Quantitative RT-PCR analysis indicates a progressive increase in *RUNX1* expression with a corresponding decline in *MET*/c-Met indicating acquisition of non-peptidergic nociceptor fate. Gene expression levels were normalized to *GAPDH* and are presented as values relative to a sample of undifferentiated iPSC (i.e. Day 0). (**E**) Expression of the non-peptidergic marker, RET, in mature neurons. (**F**) RT-qPCR detection of *KCNK18* (TRESK) mRNA transcript level increases as nociceptors mature (*P > 0 .05, one‐way ANOVA). *GAPDH* expression was used as a housekeeping gene to normalize expression levels. (**G**) Whole cell recording of single action potentials can be evoked reliably in both control nociceptors and F139WfsX24 nociceptor lines. (**H**) Representative voltage clamp data showing inward and outward currents recorded during step membrane depolarization of control wild-type (AH017-7) and F139WfsX2 (839) nociceptors. Unless otherwise stated, scale bars = 50 μm.

IPSC lines were differentiated into peripheral nociceptors by dual SMAD inhibition for neuralization, followed by GSK3β, VEGF and Notch inhibition for neural induction and subsequently matured with growth factors NGF, GDNF and BDNF for 60 days to result in homogenous cultures of electrophysiologically mature nociceptors (Fig. 1). As the cells differentiated there was progressive loss of pluripotency markers OCT-4 and acquisition of neural crest marker P75^NTR^ by Day 8 (Fig. 1A and B). Maturation resulted in pure populations of Brn3a^+^ sensory neurons (Fig. 1C). Through early differentiation, c-Met was transiently expressed while expression of the transcription factor RUNX1 was delayed but sustained, consistent with *in vivo* development of non-peptidergic nociceptors (Fig. 1D). Furthermore, neurons strongly expressed the non-peptidergic marker, Ret at Day 50 (Fig. 1E).

*KCNK18* mRNA expression was absent in iPSC but steadily increased during nociceptor differentiation and maturation (Fig. 1F). All lines differentiated efficiently into nociceptors, yielding 24–49% of neurons expressing Islet-1 at Day 14 in culture (Supplementary Fig. 1F). There were no significant differences in yield between the iPSC lines, and all of them were able to fire action potentials reliably and displayed intrinsic neuronal properties of mature neurons (Fig. 1G and H).

### TRESK F139WfsX24 nociceptors show increased neuronal excitability associated with a loss of TRESK function

**Figure 2:**
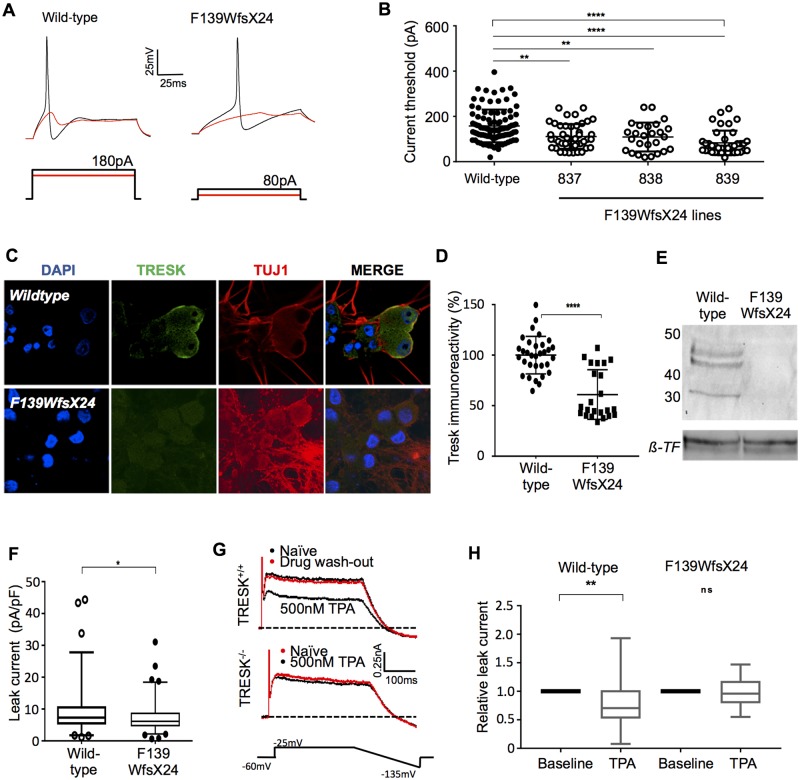
**Comparison of control and F139WfsX24 mature iPSC‐derived nociceptors.** (**A**) Representative current clamp traces, showing subthreshold responses and subsequent action potentials evoked until reaching current threshold (rheobase) of 220 pA for wild-type (NHDF-1) and 90 pA for F139WfsX24 cells (Line 839). (**B**) All three cell lines carrying the F139WfsX24 mutation required significantly lower rheobase than wild-type (one-way ANOVA, *n* total = 298, *P* < 0.0001, mean rheobase ± SEM: wild-type 158 pA ± 7.4; F139WfsX24 line 837 111.5 pA ± 8.2; line 838 109 pA ± 12.5; line 839 83.4 pA ± 8.7) (**C**) Representative images illustrating the presence of TRESK in Day 50 iPSC-derived nociceptors from wild-type or F139WfsX24 lines (**D**). Image intensity analysis showed reduced TRESK expression in F139WfsX24 migraine nociceptors (Mann-Whitney test, *P <* 0.0001). TRESK was present in cell lysate surface membrane fractions of wild-type nociceptors, but absent in F139WfsX24 nociceptors by western blot (**E**). β-Transferrin (β-TF) was used as a loading control. (**F**) F139WfsX24 migraine nociceptors had reduced potassium leak current compared to wild-type, Mann-Whitney test *P =* 0.0471. Boxplot 5-95 percentiles. (**G**) Treatment of mouse primary DRG cultures with TRESK inhibitor, 0.5 µM TPA, selectively blocks potassium leak current in wild-type DRG cultures, but has no effect on DRG cultures from TRESK knockout mice. (**H**). Similarly, 0.5 µM TPA has no effect in F139WfsX24 migrane neurons (paired *t*-test, mean reduction; wild-type nociceptors 23%; F139WfsX24 nociceptors 2.4%). Boxplot 5–95 percentiles. DRG = dorsal root ganglia.

**Figure 3:**
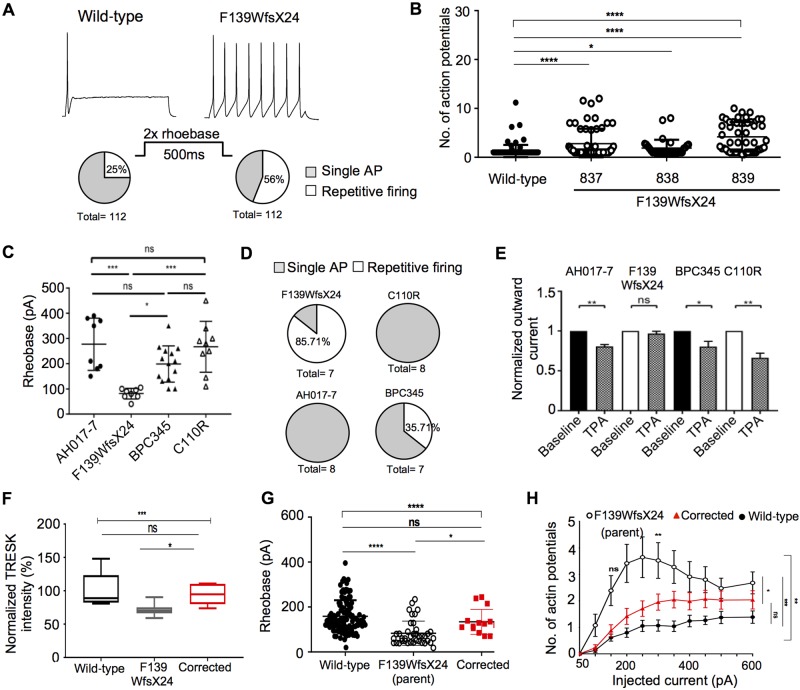
**F139WfsX24 causes neuronal hyperexcitability in iPSC-derived nociceptors.** (**A**) Injecting a suprathreshold current into neurons resulted in increased repetitive firing in F139WfsX24 lines (56% versus controls 26%, Fisher’s exact test *P <* 0.0001). Representative trace from NHDF-1 (control) and 839 (F139WfsX24) (**B**) The number of action potentials generated from a suprathreshold stimulus (2× rheobase) were quantified and observed to be significantly higher in all three F139WfsX24 migraine iPSC nociceptors (one-way ANOVA, *P* < 0.001, *n* = 136). (**C**) IPSC lines were reprogrammed from erythroblasts from subjects with the C110R variant (8152) or wild-type (BPC345) and compared to fibroblast derived iPSC from wild-type (AH017-7) or F139WfsX24 (839). The F139WfsX24 nociceptors show a significantly reduced rheobase compared to wild-type or C110R variant nociceptors. (**D**) The C110R nociceptors did not fire repetitively to a suprathreshold stimulus unlike the F139WfsX24 nociceptors and (**E**) TPA was able to inhibit outward leak current in wild-type (19.13%) and C110R (33.46%) nociceptors, but had minimal effect in the F139WfsX24 nociceptors. (**F**) F139WfsX24 iPSC line (839) was genome ‘corrected’ to the wild-type allele and TRESK expression recovered to wild-type levels [Kruksal-Wallis test *P =* 0.004, Dunn’s multiple comparisons test: wild-type versus F139WfsX24 *P* = 0.0009, wild-type versus correction, not significant (ns), F139WfsX24 versus correction *P* = 0.0103]. Boxplot 5–95 percentiles. (**G**) Genome-corrected iPSC neurons, reversed rheobase to wild-type levels (one-way ANOVA *P <* 0.0001; Dunn’s multiple comparisons; wild-type versus parent F139WfsX24 *P <* 0.0001, wild-type versus corrected ns, corrected versus parent F139WfsX24 *P <* 0.05). (**H**) Increasing suprathreshold current injection, induces a progressive increase in the number of action potentials fired by parent F139WfsX24-nociceptors compared to both wild-type and genome corrected clone (one-way ANOVA, ^**^P = 0.0099, with Sidaks multiple comparison wild-type versus parent F139WfsX24 *P <* 0.001, wild-type versus corrected ns, corrected versus parent F139WfsX24 *P <* 0.05). Data represent mean ± SEM pooled from three independent differentiations.

Patch clamp recordings demonstrated a reduced rheobase of TRESK F139WfsX24 nociceptors compared to control (F139wfsX24 101 ± 5.5 pA versus healthy control 158 ± 3.4 pA), indicative of hyperexcitability (Fig. 2A and B). Furthermore, injecting a suprathreshold stimulus, the healthy control neurons typically fired a single action potential, whereas 56% of TRESK F139WfsX24 neurons fired repetitively (Fig. 3A, *P <* 0.0001 Fisher’s exact test). TRESK F139WfsX24 nociceptors also fired a greater number of action potentials to suprathreshold stimuli (Fig. 3B). Comparing the two groups, there were no significant differences in the resting membrane potential (RMP) (healthy control −58.79 ± 0.89 mV, F139WfsX24 −58.16 ± 1.00 mV, *P >* 0.05 student’s unpaired *t*-test) or input resistance (healthy control 258.38 ± 14.79 MΩ, F139WfsX24 260.26 ± 9.12 MΩ, *P >* 0.05 student’s unpaired *t*-test). We cultured healthy control and TRESK F139WfsX24 nociceptors on an MEA platform to investigate population neuronal activity. We found no difference in absolute firing frequency, but a significant increase in the frequency of burst activity events (at least five consecutive spikes with an interspike interval <100 ms) comparing the frameshift mutant and the control (*P <* 0.001, Supplementary Fig. 3). These data suggest that F139WfsX24 nociceptors are more likely to repetitively fire multiple action potentials once activated and are therefore congruous with the hyperexcitability observed by patch clamp recordings.

To investigate further the mechanisms by which the TRESK F139WfsX24 nociceptors have heightened excitability, we analysed the expression of TRESK (Fig. 2C–E). We used an antibody targeting amino acid residues 200–250 in the large intracellular loop (not translated in the F139WfsX24 mutant allele) to demonstrate a 50% reduction in protein by immunocytochemistry in TRESK F139WfsX24 nociceptors, consistent with the heterozygous nature of F139WfsX24 (Fig. 2D). In addition to a global reduction, we noted that TRESK protein was predominantly cytoplasmic in TRESK F139WfsX24 nociceptors and not localized with the membrane (Fig. 2C). To assess membrane expression, we performed a membrane preparation of iPSC nociceptors and a western blot for TRESK expression. We found that wild-type TRESK protein was absent in membrane fractions of F139WfsX24 nociceptors, but detectable in the healthy control (Fig. 2E and Supplementary Fig. 4). Hence the mutant channel appears to be exerting a dominant negative effect on wild-type channel to prevent wild-type channel membrane expression.

Voltage clamp recordings confirmed a functional reduction of leak potassium current in F139WfsX24 nociceptors (Fig. 2F). There was still a substantial leak current in F139WfsX24 nociceptors indicating the presence of other K2P channels. To isolate the TRESK component, we have previously used TPA applied extracellularly at 0.5 μM to selectively inhibit TRESK (Wright *et al.*, 2013). At this concentration, in mouse primary trigeminal ganglion neurons, we found that TPA reduces the leak current in wild-type trigeminal ganglion neurons but has no effect on trigeminal ganglion neurons with TRESK genetically ablated, indicating a selective effect of TPA (Fig. 2G). TPA had no effect on the activity of another key K2P channel, TREK-1, when expressed in HEK cells (Supplementary Fig. 5). We found that TPA reduced the leak current in healthy control cells, but had no effect on F139WfsX24 nociceptors (healthy controls 22.98 ± 10.14% versus F139WfsX24 2.41 ± 5.9%, *P =* 0.0068 Wilcoxon matched-pairs) (Fig. 2H).

### TRESK C110R nociceptors do not show increased neuronal excitability

The C110R mutation has previously been shown to have a dominant negative effect, with a profound loss of TRESK current in *Xenopus* oocytes (Andres-Enguix *et al.*, 2012). We therefore obtained erythroblasts from a blood sample from a non-headache subject with the C110R mutation and reprogrammed to iPSCs using Sendai virus. Reprogramming from adult cells to iPSCs is thought to clear the epigenetic memory and have little influence on the subsequent phenotypes of the differentiated cells (Rouhani *et al.*, 2014). However, as the previous iPSC lines were generated from dermal fibroblasts, we also reprogrammed erythroblasts from a healthy control subject without the C110R variant to have comparable controls.

Despite the previous study indicating that C110R mutations leads to loss of function, we found C110R nociceptors to have excitability measures comparable to the healthy control nociceptors, as assessed by rheobase and the number of action potentials fired upon a supra-threshold stimulus, whether derived from erythroblasts or dermal fibroblasts (Fig. 3C–E). The TRESK F139WfsX24 nociceptors had significantly reduced excitability compared to erythroblast C110R nociceptors as indicated by the rheobase and number of action potentials fired upon a suprathreshold stimulus (Fig. 3C) (F139WfsX24 92.3 ± 10.5 pA versus C110R 291.9 ± 27.4 pA, *P =* 0.0002, one-way ANOVA followed by Tukey’s multiple comparison) and number of action potentials fired (Fig. 3D, *P =* 0.0476, Fisher’s exact test; F139WfsX24 repetitive firing 80%; control repetitive firing 20%). Furthermore, the proportion of the leak current inhibited by 0.5 μM TPA in C110R nociceptors was comparable to control (Fig. 3E) [mean reduction ± SEM; wild-type (fibroblast) 19.13% ± 1.267, wild-type (erythroblast) 19.43% ± 3.769, C110R 33.46% ± 3.265, and F139WfsX24 3.14% ± 1.9. *P =* 0.0055 one-way ANOVA followed by Kruskal-Wallis test].

### The TRESK F139WfsX24 mutation increases nociceptor excitability

To provide more evidence for a role of F139WfsX24 in altering neuronal excitability, we used CRISPR-Cas9 genome engineering to correct the mutation back to the wild-type sequence (Supplementary Fig. 6). We confirmed that there were no significant karyotypic abnormalities after cloning and expanding the corrected clone. The expression level of TRESK in nociceptors was restored to control levels (Fig. 3F, one-way ANOVA *P =* 0.004). Genome corrected iPSC nociceptors had significantly reduced nociceptor excitability as demonstrated by a reduced rheobase (one-way ANOVA *P =* 0.03, Dunnett’s multiple comparisons test, Fig. 3G) and reduced propensity to fire repeatedly to a supra-threshold stimuli (Fig. 3H, one-way ANOVA, *P <* 0.05) compared to F139WfsX24 parent clone nociceptors (line 839). Interestingly we observed that injection of current between 150 and 300 pA, had the maximal effect in generating repetitive action potentials in F139WfsX24 nociceptors with significant differences in this range to both the genome corrected line and the wild-type line. The reversal of the excitability phenotype suggests that the F139WfsX24 mutation is responsible for heightened nociceptor excitability.

### The TRESK activator, cloxyquin, reduces spontaneous nociceptor firing of an *in vitro* pain model

The voltage gated sodium channel Na_v_1.7, is considered a threshold channel, boosting depolarizing inputs to reach the action potential threshold. We reasoned that TRESK should act in an opposite manner to Na_v_1.7, to dampen rather than boost depolarizing inputs. We therefore established an MEA-based assay to investigate whether a known TRESK activator, cloxyquin, was able to reverse an *in vitro* pain cellular phenotype. We used the iPSC line RCi002-A, which is derived from a patient with inherited erythromelalgia (IEM) and carries the F1449V gain-of-function mutation in Na_v_1.7, and has previously been demonstrated to exhibit spontaneous firing when differentiated into nociceptors (Cao *et al.*, 2016).

**Figure 4:**
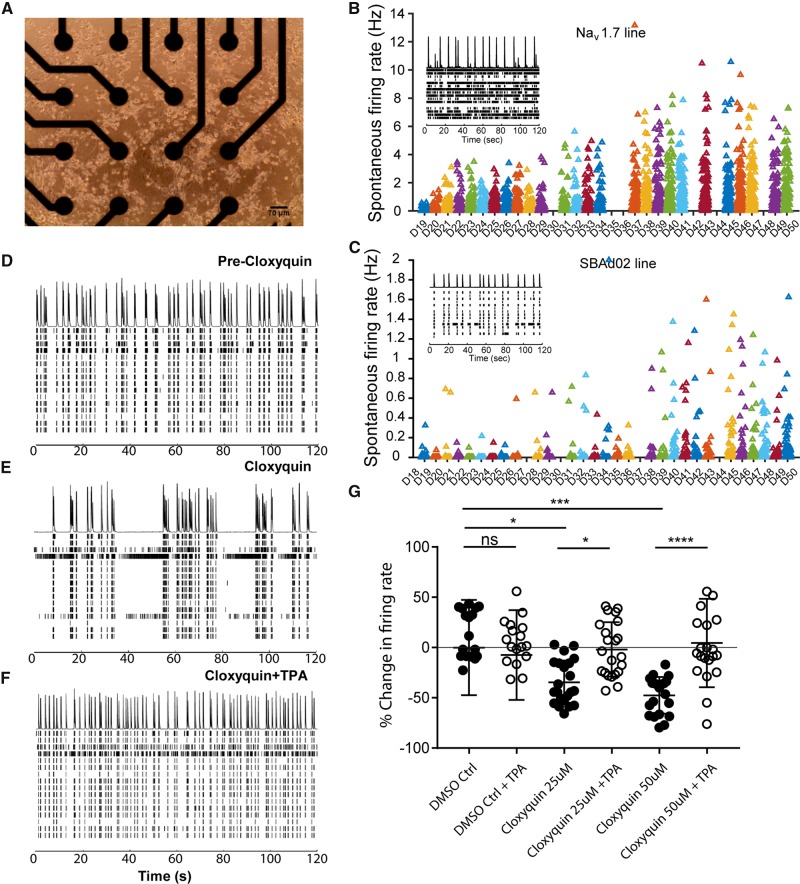
**MEA data of iPSC nociceptors.** (**A**) Representative image of density and spread of nociceptors on the electrodes in a well of 48 well MEA plate. (**B**) Recorded mean firing rate of SCN9A (Na_v_1.7) gain-of-function mutation iPSC nociceptors (*n =* 6) from Days 19 to 50 of the differentiation. Inset shows a representative raster plot and histogram of spontaneous activity of nociceptors. Each line represents recorded spike timings per electrode in the well, and the histogram at the top of the raster plot represents the sum of spiking activity. (**C**) Recorded mean firing rate of healthy control iPSC nociceptors (*n =* 6) from Days 18 to 50 of the differentiation. *Inset* shows a representative raster plot and histogram of spontaneous activity of nociceptors in a well of the plate at D50 (**D**–**F**) raster plot with histogram of spontaneous action potentials recorded in a representative well of the MEA plate. (**D**) Na_v_1.7 nociceptor response before cloxyquin. (**E**) Na_v_1.7 nociceptor response to 50 µM cloxyquin (in 0.01% DMSO); (**F**) Na_v_1.7 nociceptor response to 50 µM cloxyquin (in 0.01% DMSO) with 0.5 µM TPA. (**G**) Percentage change in firing rate of nociceptors in response to increasing dose of cloxyquin. Reversal of inhibition by subsequent addition of 0.5 µM TPA is observed. One-way ANOVA multiple comparison: *P < 0.05; ^***^P < 0.001; ^****^P < 0.0001.

RCi002 was patterned towards nociceptors, plated onto multi-well MEA plates as a co-culture with rat astrocytes and then matured for 60–70 days (Fig. 4A). The iPSC IEM mutant Na_v_1.7 nociceptors and healthy control nociceptors progressively increased their activity with maturation in culture. The IEM nociceptors showed sustained spontaneous firing, with the majority of electrodes in a well active and the mean firing rate at 1–3 Hz compared to the control nociceptors firing of <1 Hz, (Fig. 4B, C and Supplementary Fig. 7). We then added increasing doses of cloxyquin and found a significant inhibition of firing rate compared to the DMSO vehicle control [% change ± SEM: 25 µM cloxyquin −35.0% ± 4.6 (one-way ANOVA *P =* 0.0257); 50 µM cloxyquin −47.5% ± 4.0 (one-way ANOVA *P =* 0.0006); Fig. 4D–G]. Furthermore, subsequent addition of 0.5 µM TPA was able to reverse the cloxyquin effect, confirming that cloxyquin is specifically exerting its neuronal dampening through activation of TRESK.

### Cloxyquin is effective in reducing mechanical and thermal hyperalgesia in the chronic nitroglycerine model

**Figure 5:**
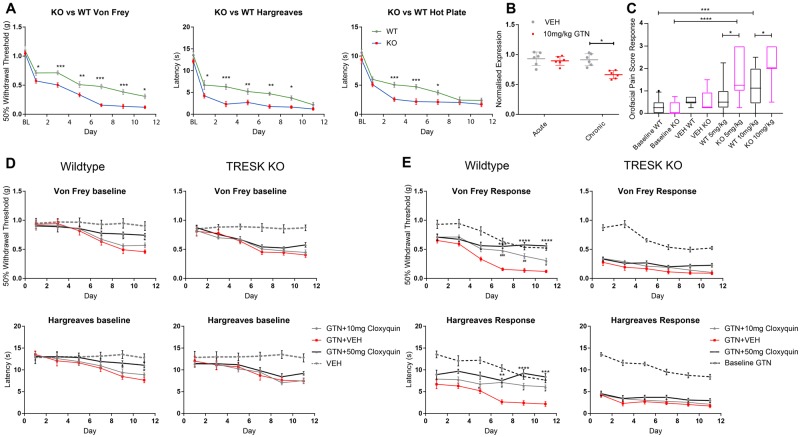
**The effect of TRESK loss or activation in the chronic GTN rodent migraine model.** (**A**) TRESK wild-type (WT) (*n =* 6) and knockout (KO) (*n =* 7) littermates were dosed daily for 11 days with 10 mg/kg GTN and mechanical and thermal sensitivity (Hargreaves test and 53°C hot plate test) was recorded 2 h post-GTN daily to obtain a measure of acute sensitization [von Frey, two-way ANOVA *F*(1,14) = 54.97, *P <* 0.0001; Hargreaves, two way ANOVA *F*(1,14) = 74.2, *P <* 0.0001; hot plate, two-way ANOVA *F*(1,14) = 29.42, *P <* 0.0001]. Tukey’s multiple comparison test ***P < 0.001, **P < 0.01, *P < 0.05 TRESK knockout versus TRESK wild-type. (**B**) Analysis of *KCNK18* (TRESK) mRNA extracted 2 h following 10 mg/kg GTN treatment on Day 1 (acute) and after 11 days of daily GTN injections (chronic). Two-way ANOVA: *F*(1,4) = 4.459, *P =* 0.0423; *P = 0.038 TRESK knockout versus TRESK wild-type. (**C**) Effects of acute GTN administration (10 mg/kg or 5 mg/kg i.p.) compared to vehicle controls in TRESK wild-type versus TRESK knockout mice on orofacial pain testing using 0.4g von Frey filament. Data are presented as mean ± SEM [VEH WT *n =* 6; VEH KO *n =* 6; GTN WT (5 mg/kg) *n =* 17 (10 mg/kg) *n =* 14; GTN KO (5 mg/kg) *n =* 7 (10 mg/kg) *n =* 7]. *P < 0.05; ***P < 0.001; ****P < 0.0001 for repeated measures one-way ANOVA with Tukey’s multiple comparison test, boxplot 5–95 percentiles. (**D**) The TRESK activator cloxyquin dose-dependently reduces baseline sensitization to von Frey filaments [two-way ANOVA: *F*(3,36) = 22.89, *P <* 0.0001; Tukey’s multiple comparison test *P < 0.05, GTN+50 mg/kg cloxyquin versus GTN+Vehicle] and Hargreaves test [two-way ANOVA: *F*(3,36) = 15.08, *P <* 0.0001; Tukey’s multiple comparison test *P < 0.05, GTN+50 mg/kg cloxyquin versus GTN+vehicle] after repeated intraperitoneal GTN in wild-type mice. No significant differences were observed in TRESK knockout mice treated with cloxyquin. (**E**) Sensitivity following acute GTN in chronically sensitized TRESK wild-type mice is significantly attenuated with 10 mg cloxyquin and to a greater degree with 50 mg cloxyquin [two-way ANOVA von Frey: *F*(3,36) = 80.57, *P <* 0.0001; Tukey’s multiple comparison test ****P < 0.0001, ***P < 0.001 GTN+50 mg cloxyquin versus GTN+vehicle; ^###^*P <* 0.001 ^##^*P <* 0.01 ^#^*P <* 0.05, GTN+10 mg cloxyquin versus GTN+vehicle; two-way ANOVA Hargreaves: *F*(3,36) = 76.4, *P <* 0.0001; Tukey’s multiple comparison test ****P < 0.0001, ***P < 0.001, **P < 0.01 GTN+50 mg cloxyquin versus GTN+vehicle; ^#^*P <* 0.05, GTN+10 mg cloxyquin versus GTN+vehicle]. No significant differences were observed in TRESK knockout mice treated with cloxyquin. wild-type *n =* 6 and knockout *n =* 9 for **D** and **E**. All data represent mean ± SEM. Baseline with GTN represents daily thresholds taken prior to GTN injection.

Having demonstrated the efficacy of cloxyquin *in vitro*, we next examined a well-established rodent migraine model (Pradhan *et al.*, 2014). Chronic intermittent GTN administration in rodents induces aversion to light in addition to thermal and mechanical hypersensitivity; features that are common among migraineurs. After a single dose of 10 mg/kg GTN intraperitoneally, wild-type mice developed hindpaw mechanical and thermal hypersensitivity, which was reversible with the migraine abortant therapy, sumatriptan (Supplementary Fig. 8A). To produce a chronic model, we injected a cohort of animals with GTN daily, and measured sensitivity on each testing day before each dose (termed ‘baseline’) and 2 h after injection (termed ‘acute’). Consistent with previous studies we found acute hypersensitivity (mechanical and thermal) following each GTN dose, in addition to a progressive increase of baseline sensitivity as treatment continued. Increases in acute sensitivity to GTN were reversed with the migraine preventative topiramate (Supplementary Fig. 8B). We used TRESK knockout mice, in which the majority of the *Kcnk18* coding region has been removed (Valenzuela *et al.*, 2003), in order to mimic the complete loss of function conferred by the F139WfsX24 mutation in an *in vivo* context and to assess the contribution of TRESK to hypersensitivity following chronic GTN. Genetic ablation of TRESK did not alter expression of TREK-1 or TREK-2, two other K2P channels recently implicated in migraine pathogenesis (Royal *et al.*, 2019) (Supplementary Fig. 9). TRESK knockout mice developed similar baseline hypersensitivity after chronic GTN (Fig. 5D); however, they exhibited persistently exaggerated mechanical and thermal sensitivity following acute GTN (Fig. 5A). The difference between the genotypes was diminished (mechanical), or lost (thermal), at later time points of chronic dosing (Fig. 5A). Consistent with this finding, we found that *KCNK18* mRNA expression was significantly reduced at a later time point (∼30% reduction, *P* = 0.038; Fig. 5B). We used mechanical testing of the periorbital facial region to assess orofacial pain behaviours following acute GTN. Wild-type and knockout animals treated with 10 mg/kg GTN both demonstrated enhanced responses compared to vehicle-treated control (Fig. 5C). However, following treatment with a lower dose of GTN (5 mg/kg), only TRESK knockout animals demonstrated enhanced responses to baseline (Fig. 5C), demonstrating that mice lacking TRESK have heightened susceptibility to GTN-induced mechanical sensitization of the orofacial region.

Wild-type mice treated with 50 mg/kg cloxyquin demonstrated reduced baseline sensitization after repeated GTN injections (Fig. 5D). Furthermore 50 mg/kg cloxyquin was able to completely prevent acute responses to GTN, following chronic intermittent GTN administration (Fig. 5E). This effect was dose dependent, as 10 mg/kg cloxyquin was non-effective in reducing baseline sensitization and less effective in preventing acute GTN responses (Fig. 5D and E). In TRESK knockout mice, cloxyquin had no discernible effect on baseline sensitization, or the acute sensory hypersensitivity induced by GTN in chronically dosed animals (Fig. 5D and E). These findings support the role of TRESK in migraine development and its potential promise as an analgesic drug target.

## Discussion

The cellular substrates of migraine are poorly understood but activation of the trigeminovascular system, with trigeminal nociceptors innervating dura blood vessels, is likely a prerequisite for the painful headache phase (Cader, 2013). Therefore any factor that results in increased excitability of nociceptors may increase the likelihood of converging processes such as cortical spreading depression or other neurogenic signals to more easily activate nociceptors and trigger headaches. TRESK is an important ion channel in nociceptive neurons and we found that human iPSC nociceptors increasingly express functional TRESK as they mature, supporting an important role for TRESK in the maturation of intrinsic neuronal properties.

We have shown that the F139WfsX24 frameshift mutation causes hyper-excitability through a dominant negative effect of the mutant allele on the wild-type to prevent the wild-type channel from reaching the plasma membrane. It is possible the truncated protein produced by the frameshift mutant allele is able to trap the wild-type protein to prevent trafficking to the membrane. Despite the loss of TRESK protein and current at the membrane, there is no significant change in the RMP, which is consistent with prior studies showing TRESK is usually in a closed state at the RMP, so may not contribute to leak currents that maintain RMP. Furthermore, it is now appreciated that K2P channels exhibit voltage-dependency (Schewe *et al.*, 2016) and produce a large rectifying conductance at subthreshold potentials to inhibit neuron firing. Additionally, they are responsive to a range of intracellular signals including G-protein subunits and uniquely in the case of TRESK, responding to calcium (Czirjak *et al.*, 2004). Hence an absence of TRESK would remove the ‘brakes’ from pain initiating signals that activate nociceptors.

Our findings that TRESK effectively acts as a brake upon depolarizing stimuli that may trigger action potential firing in nociceptors raised the possibility that TRESK activators could be effective pain therapeutics. Patients with gain of function mutations in the *SCN9A* gene, encoding the voltage gated sodium channel, Na_v_1.7, have IEM and experience chronic extreme pain (Dib-Hajj *et al.*, 2005). Na_v_1.7 is considered a threshold channel, to boost depolarizing inputs below action potential threshold. *SCN9A* gain-of-function mutations alter the channel biophysical properties, such that they are more likely to be activated at hyperpolarizing potentials and therefore have an increased liability to fire action potentials. Nociceptors generated from patients with IEM display increased excitability with reduced rheobase and increased spontaneous nociceptor firing and this *in vitro* phenotype is sensitive to Na_v_1.7 blockers including a clinical test agent (Cao *et al.*, 2016). As TRESK is also a ‘threshold’ channel but acts in an opposite manner to Na_v_1.7, TRESK activation should be able to counter the increased liability to firing action potentials in the IEM pain model. We used cloxyquin as a TRESK activator, a drug that we previously identified from a drug screen of FDA approved compounds and confirmed that this led to a reduction in nociceptor firing by 50%. Cloxyquin selectively activates TRESK and not other K2P channels (Lengyel *et al.*, 2017). To demonstrate that the effect of cloxyquin was through TRESK further, we used the TRESK selective inhibitor, TPA, to show that the cloxyquin induced reduction in firing was fully reversed by TRESK blockade.

To build on our findings at the cellular level, we assessed the contribution of TRESK to sensitization in an *in vivo* migraine mouse model. Given that TRESK is highly enriched in the sensory ganglia of rodent and humans (Lafreniere *et al.*, 2010; Ray *et al.*, 2018), we focused our efforts on peripheral mechanisms. Mice lacking TRESK demonstrate exaggerated acute mechanical and thermal hyperalgesia in response to GTN treatment. These results are highly congruous with a recent study demonstrating that TRESK knockout mice exhibit exaggerated nocifensive behaviours during an inflammatory headache model (Guo *et al.*, 2019). Conversely, cloxyquin can prevent basal and acute GTN sensitization. Interestingly the effect of cloxyquin is evident despite a reduction in *KCNK18*/TRESK mRNA expression after chronic GTN treatment. Nevertheless we confirmed that cloxyquin is mediating its effects through TRESK, since cloxyquin was completely ineffective in the TRESK knock-out animals. The ability of cloxyquin to prevent acute responses to GTN after chronic sensitization may suggest that novel potent TRESK activators may remain effective as a migraine treatment in chronic migraine states. This is an important consideration since clinical experience is often that in chronic migraine, patients become refractory to the usual treatments.

Our data also highlight the challenges to investigating genetic associations between *KCNK18* variants and migraine, as any association analysis requires robust selection of only those mutations that have deleterious effects in relevant human cells. The genomic background contributes to excitability and therefore is an important consideration of mutation penetrance. A recent study highlighted the role of the polygenic risk burden even in Mendelian-like migraine families (Gormley *et al.*, 2018). The ExAC browser is also often used to assess whether a variant has population characteristics to suggest a pathogenic role such as a very low allele frequency. However, it may be less useful in the case of *KCNK18* variants because a higher allele frequency may be anticipated for two reasons: (i) a complete loss of TRESK function has clearly been tolerated in this family without affecting survival or reproductive fitness, so deleterious TRESK variants will not be subject to negative selection pressures; and (ii) the lifetime migraine prevalence of 15% or more is such that if a *KCNK18* mutation was associated with migraine, individuals with such a mutation will be in the ExAC cohort at higher frequencies than for other less prevalent disorders. Unfortunately, migraine status is not available in ExAC to investigate this further.

The identification of the C110R mutation, which shows a similar dominant loss-of-function to F139WfsX24 in heterologous expression systems and is present in healthy control subjects, significantly undermined the case that TRESK mutations may be causative of migraine. We found, however, that in human nociceptors, the C110R mutation had no loss of TRESK current compared to wild-type controls and no consequent change in nociceptor excitability. This is consistent with a previous study where TRESK wild-type, F139WfsX24 and C110R variants were overexpressed in primary mouse trigeminal neurons and only F139WfsX24 led to increased neuronal excitability (Guo *et al.*, 2014). Hence it seems only specific TRESK mutations are able to lead to a sufficiently large loss of TRESK function in human nociceptors, presumably through trapping wild-type channels and preventing membrane expression.

A recently published study describes a second mechanism by which F139WfsX24 renders nociceptors hyperexcitable (Royal *et al.*, 2019). Their study and ours complement each other and strongly support the hypothesis that F139WfsX24 plays a causal role in migraine pathogenesis. Royal *et al.* performed elegant experiments to discover a second translational start site induced by the frameshift mutation that results in the production of two transcripts (termed MT1 and MT2). When overexpressed in heterologous expression systems MT1 acts in a dominant negative manner on wild-type TRESK, whereas MT2 does so on two other K2P channels: TREK-1 and TREK-2. The authors subsequently demonstrate that MT2 is capable of enhancing trigeminal ganglion excitability and that when overexpressed *in vivo*, results in orofacial mechanical hypersensitivity. Our data are consistent with their findings and it is possible that downregulation of TREK-1/2 channels contributes to the hyperexcitability we observed in F139WfsX24 iPSC-nociceptors. However, our finding of greater inhibition of wild-type TRESK currents by F139WfsX24 versus C110R TRESK and the phenotype of TRESK knockout mice treated with GTN, strongly suggest that loss of wild-type TRESK conductance is a major pathological contributor in itself. TRESK expression is restricted to select populations of trigeminal neurons (Weir *et al.*, 2019). Future studies should assess TRESK/TREK co-expression and also whether MT2 expressed at endogenous levels in human nociceptors results in TREK-1/2 inhibition and the relative contribution made by down-regulation of wild-type TRESK and TREK-1/2 to migraine pathogenesis. This could be addressed by generating a F139WfsX24 TRESK knock-in mouse model, or by overexpressing MT1 and MT2 in TRESK knockout and TREK-1/2 knockout transgenic mice.

Our data therefore support a genetic role of specific TRESK mutations in migraine. Moreover, we also demonstrate using *in vitro* and *in vivo* models that TRESK activators are a promising therapeutic approach to pain and migraine.

## Supplementary Material

awz342_Supplementary_Data

## Abbreviations

GTN =: nitroglycerine
iPSC =: induced pluripotent stem cell
K2P =: two-pore domain K^+^
MEA =: multi-electrode array
TPA =: tetrapentylammonium bromide
