# Supplementary material for: A causal role for TRESK loss of function in migraine mechanisms
Source: Brain. 2019 Nov 19;142(12):3852–67. doi: 10.1093/brain/awz342 (PMC6906598; doi:10.1093/brain/awz342)
Supplement: awz342_Supplementary_Data [file awz342_supplementary_data.pdf]

Supplementary Data

Supplementary Table 1: iPSC lines

iPSC name (this paper)	Full iPSC clone name(s) and previously published reference	Age and sex	Disease status	KCNK18 genotype	Reprogrammed from
NHDF1	iPS-NHDF-1(6)	44, F	Control	Wildtype	Dermal fibroblasts
AH017	AH017-7(7)	67, F	Control	Wildtype	Dermal fibroblasts
SBAD02	SBAD2-03	51, M	Control	Wildtype	Dermal fibroblasts Lonza 24245
BPC345	BPC345	XX, M	Control	Wildtype	Erythroblasts
837	837-06	65, F	Migraine	F139WfsX24	Dermal fibroblasts
838	838-03	64, F	Migraine	F139WfsX24	Dermal fibroblasts
839	839-04	63, F	Migraine	F139WfsX24	Dermal fibroblasts
8152	BP8152	71, M	Control	C110R	Erythroblasts
RCi002	RCi002-A	46, F	Inherited Erythromelalgia	KCNK18: wildtype<br>SCN9A :F1449V	Dermal fibroblast

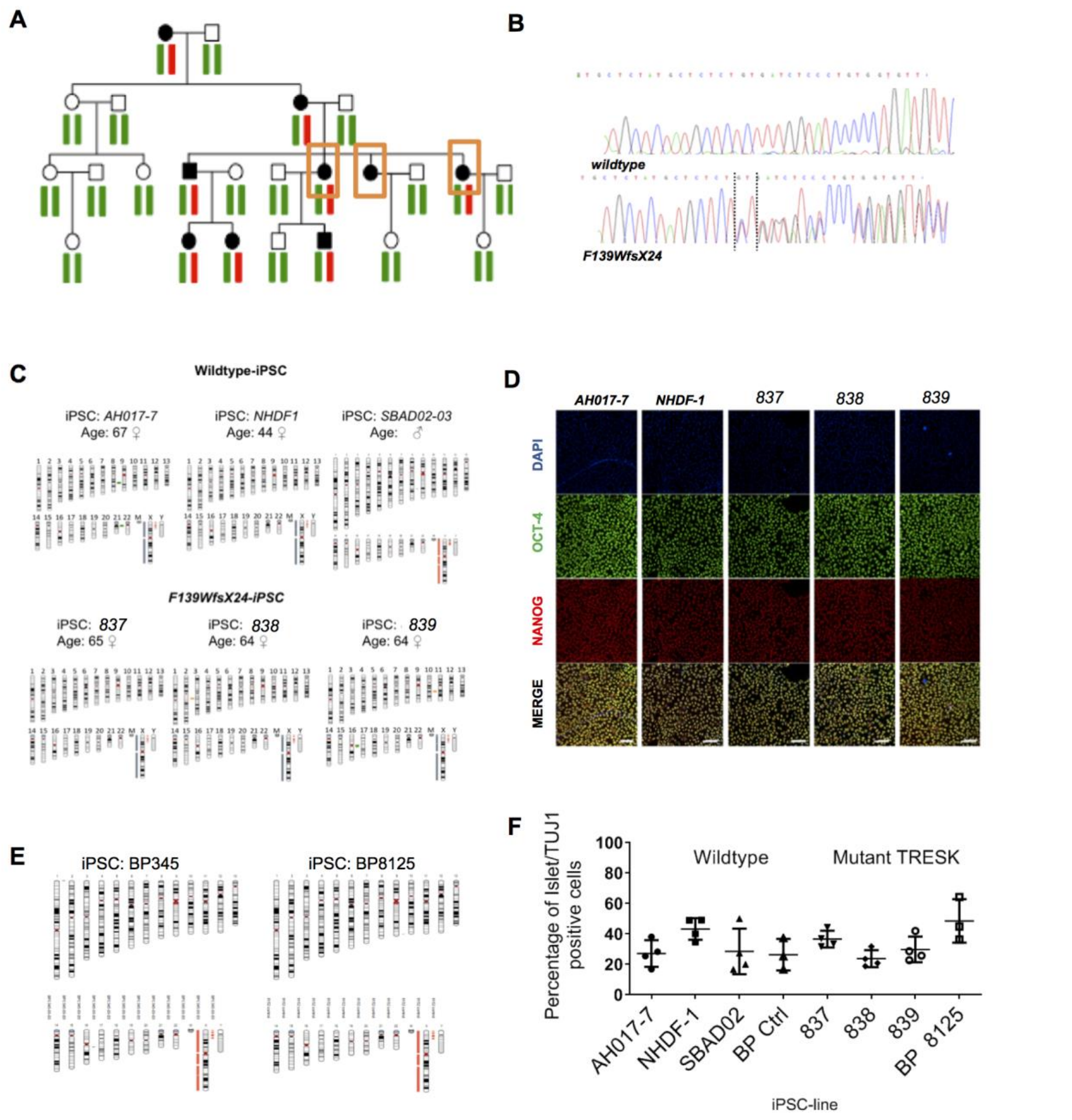

Supplementary Figure 1: iPSC line characterization. **(A)** Migraine family with iPSC lines generated from three subjects who are sisters (orange box) **(B)** Sequencing of the KCNK18 gene demonstrates the wild-type sequence for control lines, but a 2bp CT deletion at position c.414 (marked by dashed lines) in one allele of patient iPSC lines, representing the previously reported F139fsWx24 variant. **(C)** Karyogram showing genome integrity of iPSC (Illumina SNP array data analyzed using Karyostudio). Autosomal detected regions deviating from reference data are annotated with green (amplification) or orange bands (deletion) **(D)**: Human iPSC are positive for pluripotency markers OCT-4 (green) and NANOG (red); scale bar = 100 µm. **(E)** Karyogram showing genome integrity of

iPSC derived from erythroblasts **(F)** Quantification of TUJ1 and Islet1 positive cells shows 24%–43% of differentiating cells are sensory neurons, with non-significant differences between the genotypes ($p > .05$, one-way ANOVA).

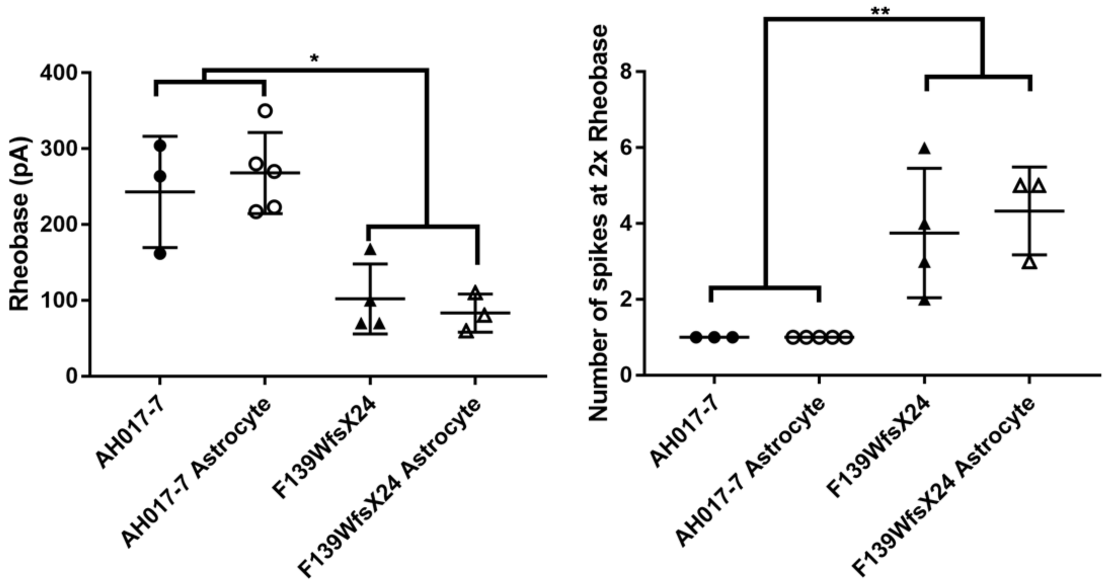

Supplementary Figure 2. Characteristics of F139WfsX24 remains the same with or without Astrocyte co-culture. Electrophysiology patching experiment on AH017 (n=3), AH017 with rat astrocytes (n=5), F139WfsX24 (n=3), F139WfsX24 with rat astrocyte (n=3) showed the same difference as was observed with previous patching experiments. * p<0.05, ** p<0.005 by one-way ANOVA with Tukey's multiple comparison test; all data presents mean $\pm$ S.D.

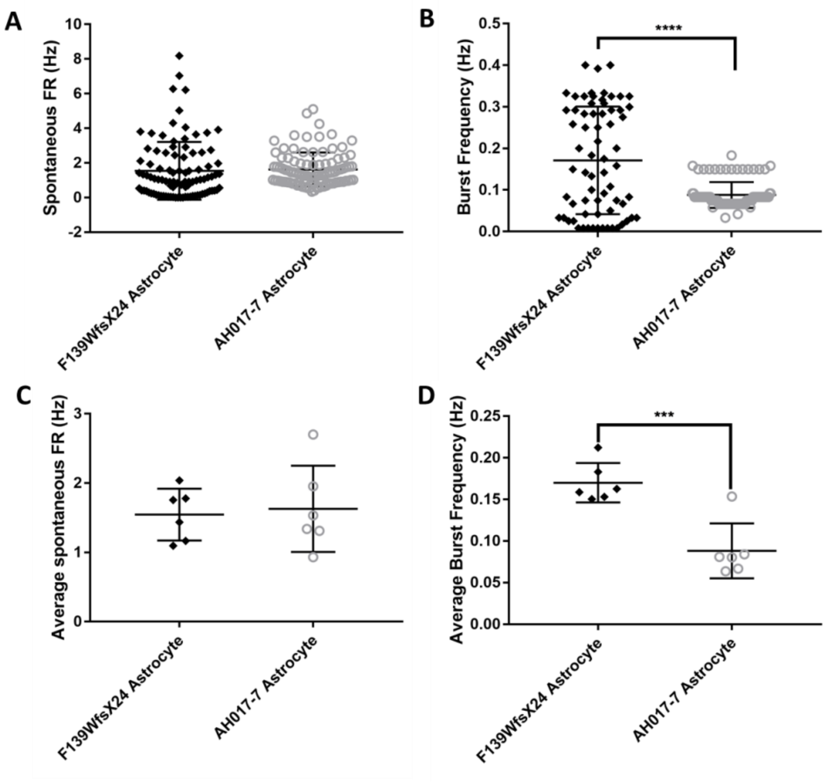

Supplementary Figure 3. Neuronal activity of F139WfsX24 Vs healthy control on MEA. (**A-B**) Spontaneous firing rate (Hz) and burst frequency (Hz) of F139WfsX24 (n=96 electrodes) and healthy control nociceptors (n=96 electrodes). Data pooled from 6 separate wells per group (**C-D**) Same data as A and B, illustrated as data points that each represent one well. *** $p<0.001$, **** $p<0.0001$ by unpaired t-test comparison; all data presents mean $\pm$ S.D.

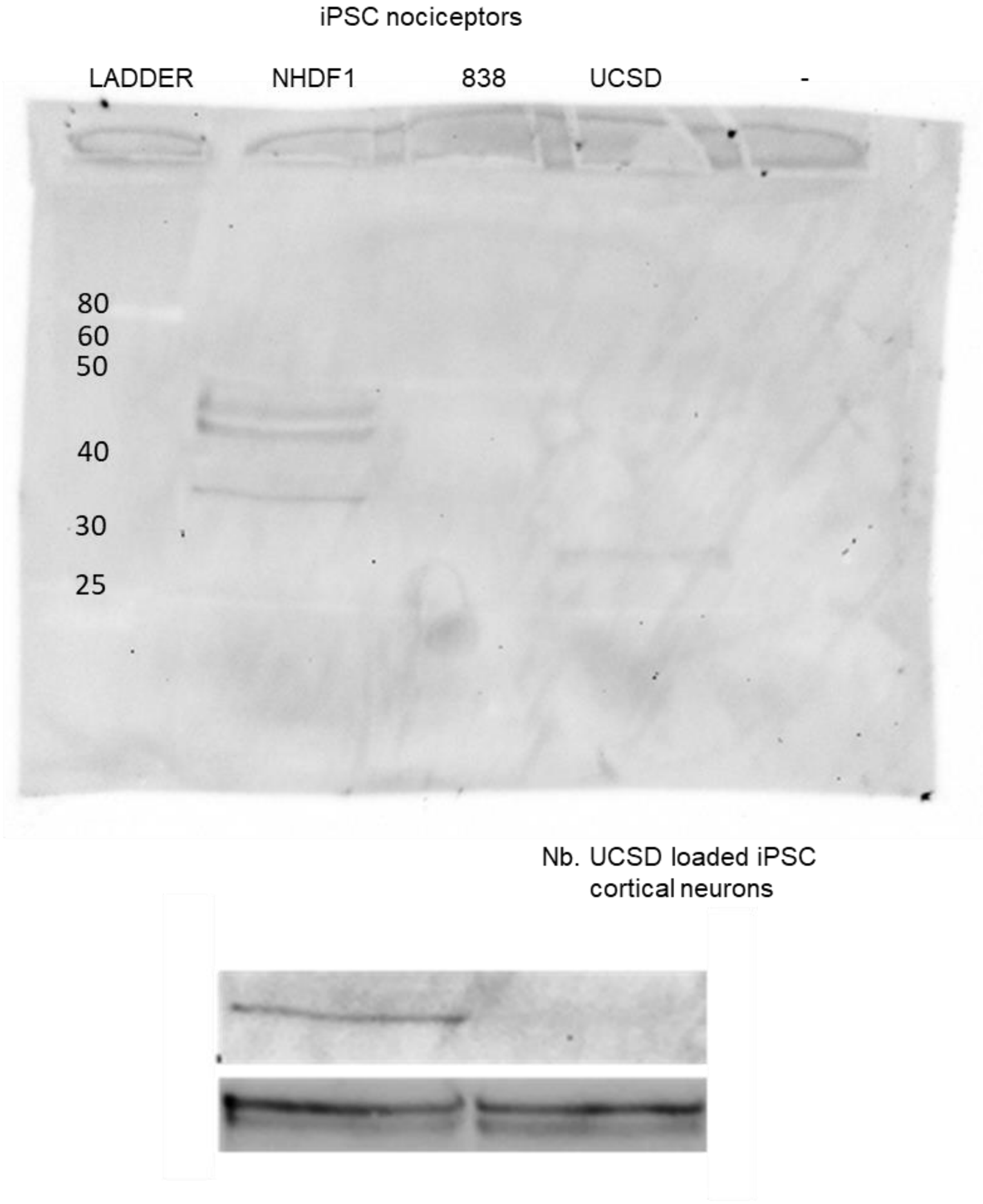

Supplementary Figure 4: Complete western blot picture for reference to Figure 2 in main document.

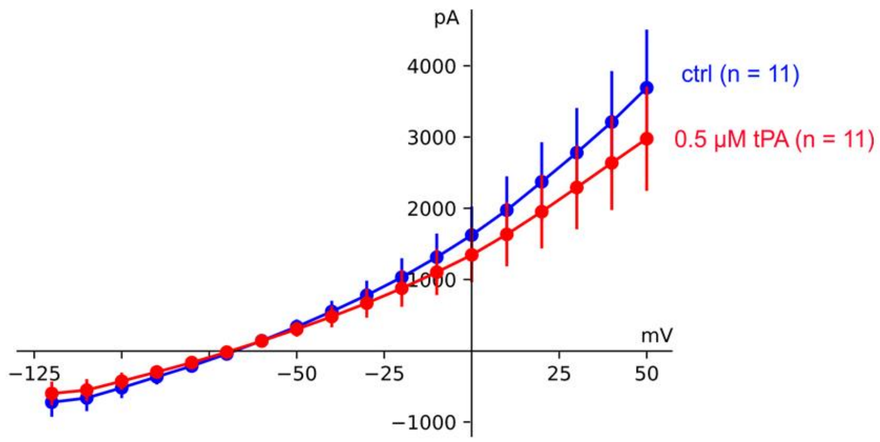

Supplementary Figure 5. HEK cells transfected with TREK1. IV curve of naive (blue) and 500nM TPA-treated (red) TREK1 current. No statistical difference was found between the two groups (two tailed *t*-test). Error bar shows ± S.D.

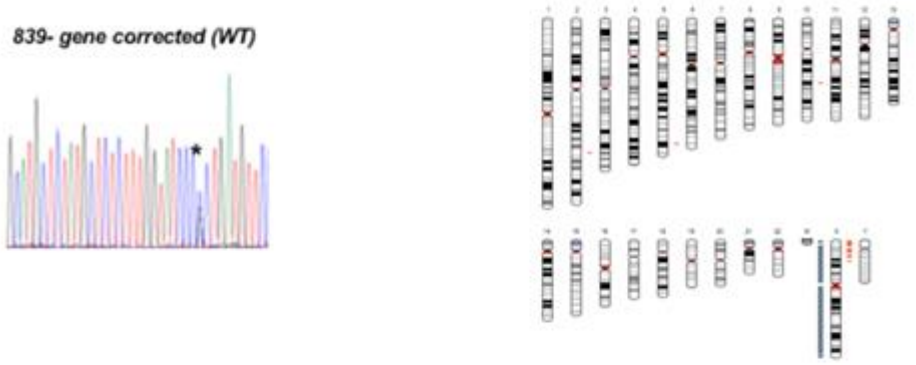

Supplementary Figure 6: Sequencing of the KCNK18 gene Gene-editing after CRISPR-Cas9 gene correction, shows gene correction of F139fsWx24 to wildtype sequence in 839 line and the corresponding Karyogram indicating no abnormalities in genome integrity have occurring during the gene-editing process.

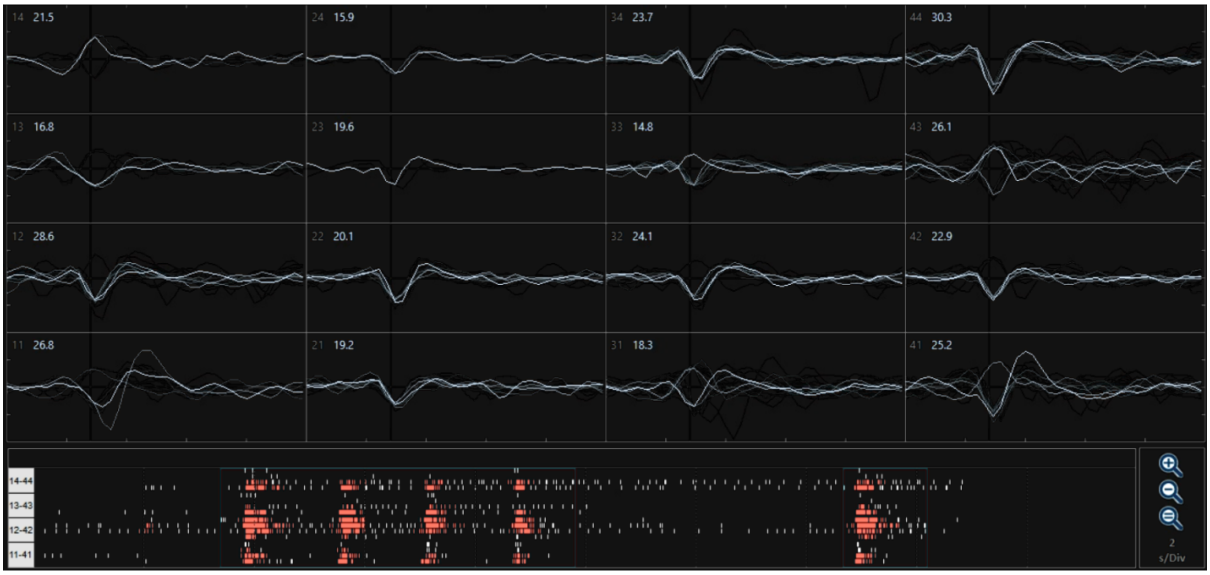

Supplementary Figure 7: Display interface of the MEA recording software showing activity recording of a well on a 48-well MEA plate. The grid graphs at the top shows the extracellular waveform recorded per electrode in this well. The raster plot at the bottom records the timing of when the extracellular spike occurred for all the electrodes in the well. Recording is organised with activity per single electrode per horizontal line. The red colouring in the graph shows where burst activity occurred.

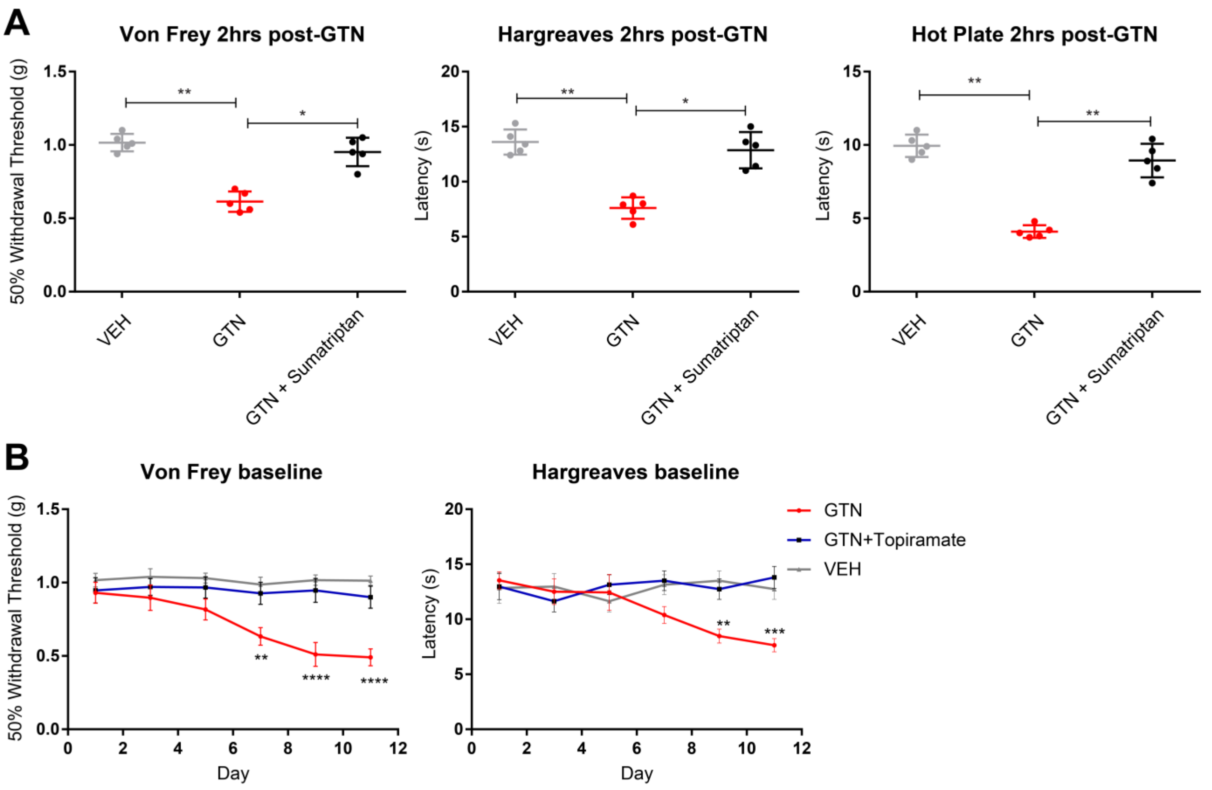

Supplementary Figure 8. Chronic GTN induces reversible mechanical and thermal hypersensitivity. (**A**) Wildtype (WT) C57BL/6J mice (n=10) injected with a single I.P. dose of 10mg/kg GTN, show significant mechanical and thermal hypersensitivity, reversible with sumatriptan when assessed using von Frey filaments ($F (2, 6) = 14.15$, p=0.0054 GTN *vs* GTN+sumatriptan; p=0.0056 GTN-only vs control), the Hargeaves test ($F (2, 6) = 15.04$, p=0.0046 GTN *vs* GTN+sumatriptan; p=0.0057 GTN-only vs control) and 53°C Hot plate test ($F (2, 6) = 21.71$; p=0.0018 GTN *vs* GTN+sumatriptan; $p = 0.0019$ GTN-only vs control). All comparisons were made using a one-way ANOVA followed by Tukey's multiple comparisons test. (**B**) Following repeated I.P. 10mg/kg GTN injections, wildtype mice demonstrate reversible mechanical (Von Frey, two-way ANOVA: $F (2, 24) = 79.35$, $p<0.0001$ followed by Tukey's multiple comparison test, ****p<0.0001 **p=0.0014 GTN *vs* GTN+topiramate) and thermal hypersensitivity at baseline (hargreaves, two-way ANOVA: $F (2, 24) = 14.98$, $p<0.0001$ followed by Tukey's multiple comparison test, ***p=0.0001 **p=0.0093 GTN *vs* GTN+topiramate). All data represents mean ± s.e.m.

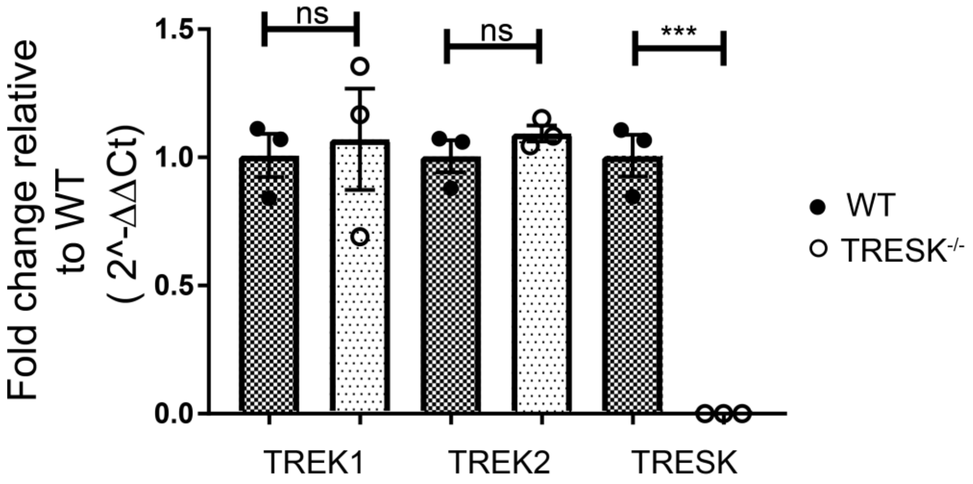

Supplementary Figure 9. mRNA transcript levels of TREK1/2 and TRESK in the DRG of wildtype and TRESK knockout mice, as measured by qPCR. No significant difference was observed for TREK1/2 expression between groups, whereas TRESK expression is not detected in the knockout tissue. * p<0.05, ** p<0.005, one-way ANOVA followed by Tukey's multiple comparison test; data represents mean $\pm$ S.E.M of 3 biological samples per group, each derived from 3 technical replicates.
